# D-dimers in omicron versus delta: A retrospective analysis

**DOI:** 10.4102/sajid.v37i1.484

**Published:** 2022-11-30

**Authors:** Alon H. Shulman, Barry Jacobson, Bradley M. Segal, Amber Khan, Jessica Trusler, Lindsay Earlam, Guy Shemesh

**Affiliations:** 1 Department of Haematology and Molecular Medicine, Faculty of Health Sciences, National Health Laboratory Service, University of the Witwatersrand, Johannesburg, South Africa; 2 Department of Medicine, Charlotte Maxeke Johannesburg Academic Hospital, Johannesburg, South Africa; 3 Clinical Pathology Division, Ampath Laboratories, Johannesburg, South Africa; 4 Department of Haematology, Lancet Laboratories, Johannesburg, South Africa

**Keywords:** SARS-CoV-2, D-dimer, B.1.1.529, B.1.617.2, omicron, delta

## Abstract

**Background:**

Infection with SARS-CoV-2 has shown to cause an increase in D-dimers, which correlate with severity and prognosis for in-hospital mortality. The B.1.617.2 (delta) variant is known to cause a raised D-dimer level, with data on D-dimers in the B.1.1.529 (omicron) variant being scarce.

**Objectives:**

To determine the effect of age, gender and SARS-CoV-2 variant on the D-dimer in South Africans admitted to tertiary medical centres from May 2021 to December 2021.

**Method:**

The study was performed retrospectively on 16 010 adult patients with a SARS-CoV-2 infection. Age, gender, SARS-CoV-2 PCR and D-dimer levels on admission were collected from two national laboratories. Admissions from 01 May 2021 to 31 October 2021 were classified as B.1.617.2, whereas admissions from 01 November 2021 to 23 December 2021 were classified as B.1.1.529 infections.

**Results:**

Omicron infections had a median D-dimer level of 0.54 µg/mL (95% CI: 0.32, 1.08, *p* < 0.001). Multivariable regression analysis showed that infection with omicron had a 34.30% (95% CI: 28.97, 39.23) reduction in D-dimer values, compared with delta infections. Middle aged, aged and aged over 80 years had D-dimer results greater than the adult baseline (42.6%, 95% CI: 38.0, 47.3, 124.6%, 95% CI: 116.0, 133.7 and 216.1%, 95% CI: 199.5, 233.3). Males on average had a 7.1% (95% CI: 4.6, 9.6) lower D-dimer level than females.

**Conclusion:**

Infection with the B.1.1.529 variant, compared with B.1.617.2 variant, had significantly lower D-dimer levels, with age being a more significant predictor of D-dimer levels, than gender and SARS-CoV-2 variant of infection.

**Contribution:**

This study provides novel insight into the hypercoagulable impact of various SARS-CoV-2 variants, which can guide the management of patients.

## Introduction

Coronavirus disease 2019 (COVID-19), caused by the severe acute respiratory syndrome coronavirus 2 (SARS-CoV-2), was first recorded in Wuhan, China, in 2019. Infection with SARS-CoV-2 causes complex and varied thrombotic abnormalities in Virchow’s Triad, which arise from endothelial injury,^1^ stasis of immobilised patients and hypercoagulable states owing to the acute inflammation, immunothrombosis and cytokine storms.^2^ Infection with SARS-CoV-2 has been shown to cause an increase in venous thromboembolic events (VTEs) with up to 14% in the total cases of COVID-19 hospitalisations, which includes an 8% rate in non-intensive care units (ICU) and a 23% rate of VTE in ICU patients.^3^

The risk and progression of VTE are measured by an increase in D-dimer levels, a fibrin degradation product, which has been widely used as a key prognostic biomarker, and mortality indicator for thrombotic disorders for in-hospital patients. A D-dimer with a value less than 0.50 µg/mL is considered normal, with normal ranges increasing with pregnancy and advancing age.^4^ The D-dimer test on admission shows promise in predicting the severity of SARS-CoV-2 infection in multiple studies.^5,6^

In South Africa, three variants including the B.1.351 (beta), which dominated the second wave from May 2020 to September 2020, with 6945 recorded cases, B.1.617.2 (delta), which dominated the third wave from May 2021 to October 2021 and accounted for 11 044 recorded infections, as well as the recently emerged B.1.1.529 (omicron), which dominated the fourth wave from November 2021 (with 431 recorded cases at the time of these results).^7^ Since the first wave, the incidences of VTE in SARS-CoV-2 patients were significantly higher in hospitalised patients in South Africa, particularly more so in the delta variant.^7^ The present study was undertaken as the patients with B.1.1.529 SARS-CoV-2 infection in Charlotte Maxeke Johannesburg Academic Hospital, a tertiary hospital in South Africa, did not present with the classically raised D-dimer levels.

Given the importance of D-dimer in assisting critical care and VTE management in COVID-19, the authors retrospectively analysed the data collected from 16 010 South African patients infected with SARS-CoV-2 to determine the association of age, gender and the levels of serum-D-dimer in two different COVID-19 infection waves dominated by the delta (B.1.617.2) (May 2021 to October 2021) and omicron (B.1.1.529) (November 2021–present) variants of concern.

## Methods

### Study design and population

The present study is a retrospective record analysis involving 16 010 adult individuals (> 18 years) who tested positive for SARS-CoV-2 infection between 01 May 2021 and 23 December 2021 in South Africa. Patients were only considered to be eligible for the study if they were infected with delta (from the National Institute for Communicable Diseases of South Africa [NICD] dashboard 01 May 2021 – 31 October 2021) or omicron (from the NICD dashboard 24 November 2021 – 23 December 2021) variants and had a confirmed SARS-CoV-2 infection on nasal swab reverse transcription polymerase chain reaction (RT-PCR) and had a record of D-dimer test (rapid enzyme-linked immunosorbent assay [ELISA] test) on hospital admission. Demographic variables such as patients’ age and gender were recorded. Patients presenting with incomplete data records or absence of a D-dimer test on admission were excluded from the study. As the (normal) reference value for D-dimer level varies in an age dependent manner, age of the patient was further stratified for analysis. Patients were classified as adults (18–44 years), middle age (45–64 years), aged (65–79 years), aged 80 years and over as per the age group definition from Medical Subject Headings (MeSH).

### Primary and secondary outcome

The primary outcome was determining the D-dimer profile for the SARS-CoV-2 variants (delta and omicron) with values > 0.50 µg/mL, indicating a positive D-dimer. The secondary outcomes were to determine the effect of age, stratified by MeSH age groups: adult (18–44 years), (45–64 years), aged (65–79 years), aged 80 years and over, as well as the effect gender on D-dimer levels in SARS-CoV-2 infected individuals, in South Africa.

### Sample size calculations

All sample size estimates were performed for a significance level of 0.05 and a statistical power of 80%. Estimates were produced using the pwr package in R.^8^ No estimates exist for an expected difference in D-dimer values between SARS-CoV-2 variants and as such effect sizes were employed from Cohen.^9^ The two-sided estimate for a small effect size (h = 0.2) in the observed proportion of elevated D-dimer values is 393 patients per group, under the aforementioned significance and power thresholds. The sample size estimate for multiple linear regression using six degrees of freedom with a similarly small effect size (f2 = 0.02) is 681 patients per group.

### Data collection

Data were received from Ampath and Lancet laboratories (South Africa) with the results of all adult individuals who had received both a COVID-19 polymerase chain reaction (PCR) test and a quantitative D-dimer ELISA. Information pertaining to the date of admission or first clinical contact was to include only those patients with a positive PCR result performed within 24 h of a quantitative D-dimer level. Following sub-setting and removal of duplicates, a total of 16 010 patient results were available. The likely causative COVID-19 variant was inferred according to surveillance figures from the NICD at the time of infection.^7^ Cumulative daily South African national vaccination rates were obtained from the Our World In Data (OWID) COVID-19 data set^10^ and were matched to the PCR test date for each patient. D-dimer values were binarised into normal and elevated according to the laboratory standard cut-off of 0.5 µg/mL.^11^

### Statistical analysis

All statistical analyses were performed within the R programming environment.^12^ Cohort descriptions and statistical comparisons between variants were produced with the table one package.^13^ Continuous variables were compared using non-parametric tests, based on the observed distribution of recorded values or if the underlying distribution was known to not conform to a Gaussian distribution (as with D-dimer values). All tests were performed as two sided with an alpha of 0.05 used as the significance threshold. D-dimer estimate modelling was performed for both laboratory binarised and the continuous cases. In both circumstances, modelling proceeded in two phases: the first considering univariate estimates for each recorded variable and the second using a composite multivariable model. All variables were retained in multivariable modelling that were evaluated in the univariate configuration. Linear modelling in the continuous case employed a logarithmic transformation applied to the recorded D-dimer values. This was performed to improve the extent to which the collected data conformed to a Gaussian distribution, given the right-skewed nature of the raw values. These results provide a multiplicative estimate of the effect of each variable on D-dimer results. All models included the daily South African cumulative vaccination rate, matched to the date of positive PCR result, in an attempt to control for bias in the clinical response owed to an increasing vaccine coverage.

### Ethical considerations

The study was approved by the University of the Witwatersrand, Human Research Ethical Committee – Medical (M2111159). The study was carried out in line with the declaration of Helsinki and the Singaporean principles.

## Results

**FIGURE 1:**
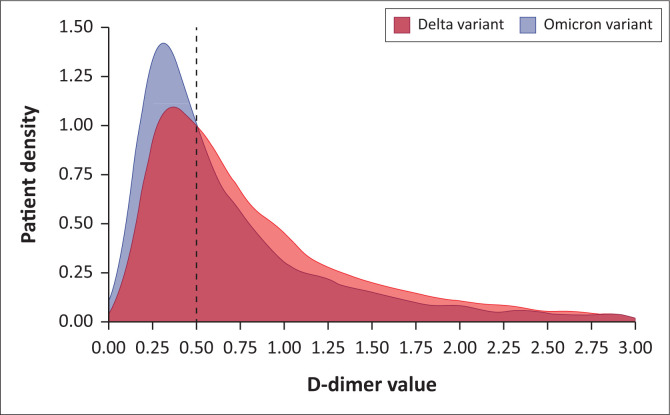
Density plot demonstrating the proportion of patients with initial D-dimer values subdivided by the most common variant of COVID-19 isolated at the time of diagnosis. The dotted line indicates the laboratory threshold for an elevated result.

Quantitatively, as well as qualitatively, D-dimer results were more substantially raised in the B.1.617.2 dominated wave cohort (*p* < 0.001). Figure 1 demonstrated the distinct D-dimer result pattern between the predominant SARS-CoV-2 variants. The pattern demonstrates that the B.1.1.529 variant-dominated wave cohort has an obvious case peak below the threshold, near 0.25 µg/mL, whereas the B.1.617.2 dominated wave cohort sustained a far longer-tailed distribution with more cases above the threshold for elevation. Furthermore, it is evident that the B.1.1.529 dominated wave demonstrates a shift in result proportions towards a near even split between thresholds. Infection with the B.1.1.529 variant demonstrated a reduction in positive D-dimer results with an odds ratio of 0.41 (95% CI: 0.33, 0.50) compared with B.1.617.2 variant cases.

The cohort of patients infected in B.1.1.529 (dominated COVID-19) wave differed in several ways to those infected in B.1.617.2 (dominated COVID-19) wave. The B.1.1.529 wave group included a greater proportion of younger individuals (*p* < 0.001), as well as having a slightly larger female preponderance (*p* < 0.001) than those infected in the B.1.617.2 dominated wave as demonstrated in Table 1.

**TABLE 1:** Study cohort stratified by the most common SARS-CoV-2 variant since diagnosis.

Features	Delta variant (*N* = 13 129)				Omicron variant (*N* = 2881)				*p*
	*n*	%	Median	IQR	*n*	%	Median	IQR	
D-dimer Value	-	-	0.70	0.41, 1.31	-	-	0.54	0.32, 1.08	< 0.001
Age	-	-	55.00	44.00, 67.00	-	-	52.00	37.00, 66.00	< 0.001
Female	7020	53.4	-	-	1676	57.9	-	-	< 0.001
Male	6124	46.6	-	-	1219	42.1	-	-	< 0.001
Age group			-	-			-	-	< 0.001
Adult (18–44 years)	3391	25.8	-	-	1098	37.9	-	-	-
Middle aged (45–64 years)	6026	45.8	-	-	997	34.4	-	-	-
Aged (65–79 years)	2677	20.4	-	-	563	19.4	-	-	-
Aged 80+ (≥ 80 years)	1050	8.0	-	-	237	8.2	-	-	-
Elevated D-dimer	8760	66.6	-	-	1553	53.6	-	-	< 0.001

IQR, interquartile range.

Sub-setting the D-dimer results by age group yielded a seemingly age-dependent effect of COVID-19 variant on initial results. The overall results demonstrate two such subsets, the first being an adult and middle-aged (64 years and younger) group that shows an increased proportion of normal D-dimer results for the B.1.1.529 variant, whereas for those 65 years and older the trend is for greater quantitative outcomes irrespective of COVID-19 variant. This relationship between age and D-dimer results is explained in Table 2. Further subdivision of this cohort by gender did not alter these age-related findings.

**TABLE 2:** Proportion of elevated D-dimer results per variant binarised according to test thresholds and sub-grouped by patient age group.

Age group (years)	Delta variant D-dimer elevation proportion	95% CI	Omicron variant D-dimer elevation proportion	95% CI	*p*
Adult (18–44 years)	45.88	44.20, 47.58	35.99	33.15, 38.93	< 0.001
Middle aged (45–64 years)	65.06	63.83, 66.26	49.24	46.09, 52.40	< 0.001
Aged (65–79 years)	85.09	83.67, 86.41	79.68	76.06, 82.88	0.002
Aged 80+ (≥ 80 years)	95.51	94.02, 96.65	91.06	86.48, 94.25	0.010

CI, confidence interval.

Age as previously demonstrated was substantially predictive of an alteration in D-dimer results. An individual over 80 years of age is estimated to have a D-dimer result 2.16 (95% CI: 2.00, 2.32) times greater than an individual within the adult (18–44 years) age group according to the multivariable model. The SARS-CoV-2 infections in the B.1.1.529 dominated wave has an estimated 34.30% (95% CI: 28.97, 39.23) reduction in D-dimer values compared with those with the B.1.617.2 wave. Results affirmed the findings of the log-linear regression model with age remaining as the most substantial predictor of elevation. Table 3 demonstrates that there is a clear positive relationship between increasing age and D-dimer values with individuals over 80 years generally accounting for greater than a 200% increase in value compared with a baseline adult. Males across this cohort demonstrate continuously lower D-dimer values compared with females, both independently and when controlling for other factors. The national vaccination rate independently produces a small reduction in the average D-dimer value, however, when controlling for other covariates instead demonstrates a small increase in D-dimer values. This reversal is likely confounded by the far more substantial difference in COVID-19 variant as the vaccination rate increased alongside the change in the dominant variant.^14^ The small elevation in D-dimer value may reflect a minor increased risk of thromboembolism in some individuals following vaccination^14^ or may be a spurious correlation with inflated significance owing to the size of sample. Table 4 focuses on a predictive model evaluating the odds ratio for returning an elevated D-dimer result for a particular patient as opposed to changes in the absolute value in D-dimer value as in Table 3. The findings are consistent with those in Table 3, producing similar effect measures for the binary case.

**TABLE 3:** Exponentiated log-linear model coefficient estimates for D-dimer values.

Demographics and SARS-CoV-2 variants	Univariate			Multivariable		
	Percentage change	95% CI	*p*	Percentage change	95% CI	*p*
Adult	-	-	-	-	-	-
Middle aged	44.2	39.65, 49.03	< 0.001	42.62	37.99, 47.26	< 0.001
Aged	124.79	116.19, 133.73	< 0.001	123.45	115.98, 133.73	< 0.001
Aged 80 years	218.36	201.62, 235.68	< 0.001	216.14	199.52, 232.34	< 0.001
Female gender	-	-	-	-	-	-
Male gender	−4.69	−7.41, -1.78	0.001	−7.13	−9.61, -4.59	< 0.001
Vaccination rate per hundred	−0.60	−0.70, -0.40	< 0.001	1.11	0.80 1.41	< 0.001
COVID-19 delta variant	-	-	-	-	-	-
COVID-19 omicron variant	−19.02	−22.04, -15.89	< 0.001	−34.30	−39.23, -28.97	< 0.001

*Source:* Adapted form Mathieu E, Ritchie H, Ortiz-Ospina E, et al. A global database of COVID-19 vaccinations. Nat Hum Behav. 2020;5:947–953. https://doi.org/10.1038/s41562-021-01122-8

SARS-CoV-2, severe acute respiratory syndrome coronavirus 2; COVID-19, coronavirus disease 2019; CI, confidence interval.

**TABLE 4:** Model odds ratios for elevated D-dimer test results.

Demographics and SARS-CoV-2 variants	Univariate			Multivariable		
	Odds ratio	95% CI	*p*	Odds ratio	95% CI	*p*
Adult	-	-	-	-	-	-
Middle aged	2.20	2.03, 2.37	< 0.001	2.14	1.98, 2.32	< 0.001
Aged	6.92	6.92, 7.74	< 0.001	7.05	6.31, 7.90	< 0.001
Aged 80 years	23.28	18.25, 30.19	< 0.001	23.44	18.36, 30.42	< 0.001
Female gender	-	-	-	-	-	-
Male gender	0.85	0.79, 0.92	< 0.001	0.76	0.71, 0.82	< 0.001
Vaccination rate per hundred	0.982	0.979, 0.985	< 0.001	1.02	1.00, 1.03	< 0.001
COVID-19 delta variant	-	-	-	-	-	-
COVID-19 omicron variant	0.58	0.54, 0.63	< 0.001	0.41	0.33, 0.50	< 0.001

*Source:* Adapted form Mathieu E, Ritchie H, Ortiz-Ospina E, et al. A global database of COVID-19 vaccinations. Nat Hum Behav. 2020;5:947–953. https://doi.org/10.1038/s41562-021-01122-8

CI, confidence interval.

## Discussion

Thrombosis is one of the leading causes of mortality worldwide with an estimate of one in every four deaths caused by thromboembolic complications.^15^ Coronavirus disease 2019 has transformed the whole perspective of the rate of VTE and has increased the total global thromboembolic risk and burden after 2019.^16^ Variants of SARS-CoV-2 have been shown to elevate D-dimer levels. Both B.1.1.7 (alpha) and B.1.351 (beta) SARS-CoV-2 variants were known to cause a significant rise in D-dimers and their depositions in vital organs and thus consequently less pulmonary hypoxia signalling before death.^5,17^

The emergence of the B.1.617.2 (delta) variant of concern has had a devastating thromboembolic effects at the greater population scale. Consequently, the highly transmissible B.1.1.529 (omicron) variant of concern was also expected to raise D-dimer levels. However, patients admitted with SARS-CoV-2 B.1.1.529 did not have raised D-dimers in a tertiary academic hospital in Johannesburg. The authors therefore compared the D-dimer values in individuals infected with SARS-CoV-2 in B.1.1.529 dominated with those infected in the B.1.617.2-dominated waves. The study estimates were produced with controls for participants’ age and gender.

In terms of age as a predictor of D-dimer values, middle-aged, aged and aged 80 years and above, all show a steady upward trend and are significant predictors of D-dimer values, when using adult age as a control. This is particularly the case with those 80 years and above, having the most significant D-dimer values, irrespective of the variant of infection. This is in keeping with similar findings that suggest age to be one of the most significant predictors for D-dimer levels, with both this study and literature values showing D-dimers increasing with escalating age.^6^

In terms of gender as a predictor of D-dimer levels, female gender was used as a control, and a multivariable comparison was performed. It was found that male gender had significantly lower D-dimer values (*p* < 0.001). This observation is significant and is contrary to the findings reported before^18^ in a smaller population (*n* = 107) that documented the effect of D-dimers in SARS-CoV-2 and in community-acquired pneumonia.

Individuals infected with the SARS-CoV-2 B.1.1.529 were observed to have a 34.30% (95% CI: 28.97, 39.23) reduction in D-dimer values, as compared with those infected with the SARS-CoV-2 B.1.617.2. Moreover, omicron cases had a substantially lower median D-dimer of 0.54 µg/mL (95% CI: 0.32, 1.08, *p* < 0.001), as opposed to the delta variant cases that had D-dimer values of 0.70 µg/mL (95% CI: 0.41, 1.31). In addition, the omicron variant showed a lower likelihood of returning an elevated D-dimer result with an odds ratio of 0.41 (95% CI: 0.33, 0.50) when compared with delta variant cases. Present findings do substantiate the clinical reports of the absence of a positive D-dimer level (*p* > 0.005) in B.1.617.2 infected individuals,^19^ this correlated to the small study cohort size (*n* = 336). To the best of our knowledge, this is the first study reporting a significant association of serum D-dimer levels with gender, age and variant of concern in SARS-CoV-2-infected individuals.

### Study limitations

The limitations of the study are threefold, the retrospective nature of the study, no clinical information was known about the patients, and the variants were not sequenced but rather implied using NICD majority dates.

## Conclusion

Individuals who were infected with the omicron SARS-CoV-2 variant, as opposed to the delta SARS-CoV-2 variant, showed far lower D-dimer values, with female gender having statistically higher D-dimers. However, the single most important factor influencing D-dimers is a patient’s age. This is particularly the case with those aged 80 years and above. This study provides novel evidence and insight into the hypercoagulable impact of various SARS-CoV-2 variants, which can be used by clinicians to guide the management of patients with SARS-CoV-2 infections in keeping with local guidelines.

### Recommendations

The given D-dimer results can be used to inform clinicians on patient presentation and insight into the hypercoagulable impact of two highly transmissible SARS-CoV-2 variants. The study recommends that future studies may use the given results and correlate this biochemical finding with patients’ clinical presentation.
